# Sustainable Husbandry?—A Comparative LCA of Three Lamb Breeding Systems in Turkey

**DOI:** 10.1007/s43615-023-00249-2

**Published:** 2023-01-12

**Authors:** Andreas Geß, Dalya Hazar Kalonya

**Affiliations:** 1 Department of Life Cycle Assessment, IABP, University of Stuttgart, Wankelstrasse 5, 70563 Stuttgart, Germany; 2 Department of Urban and Regional Planning, Pamukkale University, Denizli, Turkey

**Keywords:** LCA, Greenhouse gas emissions, Animal farming, Pasture management, Rural development, Turkey, Sustainability assessment, Land use, Sheep husbandry, Grazing sheep, Holistic grazing management

## Abstract

The agricultural sector has historically been the forefront economic sector in Turkey and is crucial for the rural sustainability and the pastures that are critical for biodiversity. However, inadequate policies and factors such as climate change and malpractices result in brittle pastures, rural–urban migration, and a declining agricultural sector. Also, pastures have been left without function and appropriated to other land uses such as quarries, energy power plants, and mines. Although the husbandry sector produces significant greenhouse gas emissions, pastures have a significant capacity of CO_2_ sequestration. In this study, Life cycle assessment (LCA) is applied to quantify the advantages and disadvantages of the transition between extensive and intensive production. The methodology presents a holistic analysis of the several impact categories and amounts of relevant products, services, and resource emissions along their life cycles. In order to assess the environmental effects of the lamb meat production, three sheep breeding systems in Turkey are evaluated. The study aims to promote a sustainable use of natural resources/assets without compromising the quality, competitiveness, or animal welfare and obtain recommendations for the future husbandry systems and rural development in Turkey. As an overall result, it can be stated that the intensification of sheep farming can lead to a decrease of greenhouse gas emissions per kg of meat. However, extensive sheep farming shows less impacts on soil acidification or eutrophication and can even be beneficial for erosion resistance or biodiversity if properly managed.

## Introduction

The interaction of husbandry systems with the environment has become increasingly important in Turkey within the national and local policy agendas, in line with climate change mitigation strategies and rural development, while putting a certain pressure on the intensive systems in terms of its effects on biodiversity, greenhouse gases, and other factors. Moreover, agro-food industries have been globally incorporated since the 1980s, which had several negative economical, ecological, and social outcomes due to the neoliberalized agricultural policies [1–3].

Some of the major outcomes were the declining agricultural sector and hard-up rural producers, which eventually triggered the rural–urban migration and conurbation. The mass-intensive husbandry of corn silages directly affected the food security [4]. As a result, consumer awareness of the quality and sustainability of the production cycle of animal food products has increased and the association of alternative agro-food systems (AAFS) emerged in the 2000s in order to sustain the livelihoods of the rural producers through direct producer–consumer relations and access to healthier food. Along with the increasing consumption concerns and public attention on the climate change, the contemporary agenda of the AAFS can give strong benefits if integrated into the husbandry sector. The sheep (and goat) production sector in particular shows great ecological potential due to its traditional character of extensive management [5].

Furthermore, lamb meat can prospectively gain relevance according to the recent debates on healthy and secure food. However, although it is accepted as a healthy meat option, it is seen that, among the red meat, sheep meat consumption per person is comparatively lower than cow meat consumption. Yet, purely sheep production as a healthier or ecologically better food alternative is not adequate since the animal husbandry sector is one of the major sources for greenhouse gas emissions from the agricultural sector.

There are three main management systems for the sheep breeding in the world: (1) extensive production of wool and meat products, (2) intensive milk production, and (3) traditional grazing on the pastures [3, 6–10]. As a result, the meat industry induces approximately 80% of agricultural-based emissions. Intensive agriculture has been encouraged to reduce CO_2_ emissions [11–14]. Hereby, the aim of the study is to assess the environmental impact of three sheep management systems in terms of global warming potential, acidification potential, eutrophication potential, and land use.

There are several examples of restorative agriculture practices on pastures in Turkey. One of them is Anadolu Meralari (Anatolian pastures), which is a Savory Network hub in Turkey that aims to reduce the effects of climate change due to greenhouse gas emissions by practicing “Holistic Grazing Management”—meaning a framework for decision-making that consider ecosystem processes [15]—and/or “Adaptive Multiple Paddock Grazing Management” as recommended in [15, 16]. Anadolu Meralari for example presents holistic grazing management on their land in Canakkale and provides training for other farmers [15]. A similar example in Turkey for extensive smart farming is the Vodafone Smart Village in Kocarli, Aydin [17].

Rural landscapes are in constant motion, and due to the inadequate rural policies along with the rural–urban migration, urbanization pressure, and loss of locally implicit knowledge, the husbandry sector today is in a declining stage [18]. However, there are several companies and brands associated with lamb products, which match the needs of modern consumers that seek both quality and safety, aim to increase the income of the rural producers, and protect the environment. It is seen that demanding sustainable food production systems contribute to prevent climate change and protect biodiversity [3, 6]. The diversity of large agricultural systems associated with semi-natural habitats and rural landscapes is also vital for other economic sectors such as agro- and ecotourism [19].

Thus, new tools are needed for re-assessing the livestock sector within a developing understanding of different production systems to enhance the capacity to provide ecosystem services such as climate change adaptation, biodiversity, and natural resource conservation. Pasture-based husbandry is promoted for a variety of functions including biodiversity and landscape protection, especially in Mediterranean bioregions, which differ from the Continental bioregions with their dominant intensive production [3, 11, 20]. It is important to underline that the pastures are crucial entities in terms of CO_2_ absorption capacity, biodiversity protection, erosion prevention, and the asset of forage crops. Thus, preventing the loss of pastures to other agricultural and non-agricultural land uses (e.g., quarries or mining) is an emergency issue in Mediterranean bioregions like Turkey [19, 20].

According to Daily (1997) [21], ecosystem services are the “conditions and processes through which natural ecosystems, and the species that make them up, sustain and fulfill human life.” Ecosystem services maintain biodiversity, and the production of ecosystem goods represents a crucial part of the human economy. Ecosystem services are the actual life support functions such as cleansing, recycling, and renewal and have many intangible and cultural benefits [21]. Ecosystem services are the benefits people obtain from ecosystems and are co-produced by the interactions between ecosystems and societies [22].

These definitions of the ecosystem services are parallel to Ostrom’s (1990) [23] common pool resources (CPRs) that we share, which are not limited solely collectively managed physical entities. The CPRs are defined as “natural or manmade resource systems that are sufficiently large as to make them costly to exclude potential beneficiaries from obtaining benefits from their use.” The CPRs are constituted by appropriators (providers and/or producers), resource systems (e.g., fishery, pasture), and resource units (e.g., tons of fish, tons of fodder) and differ from the public goods. The commons also refer the social relations based on common production, reciprocity, and cooperation, woven around the commonwealth. They may refer to the resources/assets to be exploited, a group of people (a community) who are united for their interests and/or a solidarity-based life outside of the capitalist system [23–25].

The holistic grazing management practices on pastures can ensure more CO_2_ absorption in the soil during extensive husbandry, which can be calculated by life cycle analysis (LCA) [16, 26, 27]. Holistic grazing management can improve basic ecosystem services such as water, mineral, and energy cycle by ecosystem tools such as fire (burning), fallow, grazing, and animal effect in the practicing fields (e.g., Menderes, Izmir; Biga, Canakkale). The observed outcomes reveal that the ecosystem services are revitalized, soil vitality (minerals and organic matter), and carbon absorption capacity are increased by the mobile paddock systems in these fields [16, 19].

The climate change–induced temperature increase has serious effects on the Mediterranean Basin such as precipitation changes, drought, excessive precipitation, and other factors that directly affect the agricultural production and food security. Recently, Turkey, which is located in the east of the basin, has also been facing those effects [28].

**Fig. 1:**
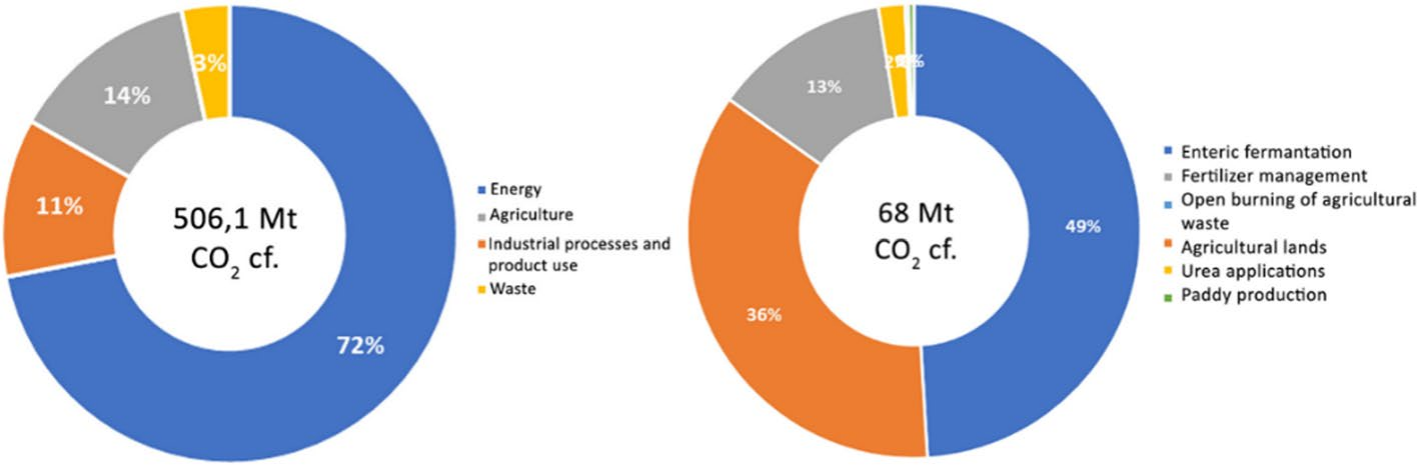
Distribution of greenhouse gas emissions by sectors in Turkey (left) (%), 2019. Distribution of greenhouse gas emissions from the agricultural sector in Turkey (right) (%), 2019 (created by the author using TURKSTAT, 2021b data [30])

According to the 5th assessment report of the IPCC, greenhouse gas emissions from waste cause 1.446 Mt of CO_2_ equivalent, 3% of total anthropogenic emissions. Since the 1970s, this figure has more than doubled [29]. The agricultural sector causes 12% of the total anthropogenic emissions, 60% of total anthropogenic nitrous oxide (N_2_O) emissions, and 50% of total methane (CH_4_) emissions in the world (see Fig. 1). Furthermore, the most important source of N_2_O is the mineral fertilizers embedded in agricultural soil [29].

Turkey’s greenhouse gas emission rates (2019) by sectors and gas types are shown in Fig. 1. Accordingly, it is seen that the agricultural sector ranks second after the energy sector with a rate of 13.4%. Looking at the distribution of gases, CO_2_ comes first with 78.9%, CH_4_ comes second with 11.9%, and N_2_O comes in third with 7.9%. When we look at the change in sectoral greenhouse gas emission rates between 1990 and 2019, it is seen that the total amount of emissions increased from 219.6 to 506.1 million tons of CO_2_ eq. While there is a regular increase in emissions originating from energy, industrial processes and product use, and agriculture sector, it is noteworthy that there has been a decrease in emissions originating from the waste sector after 2015 [30].

When the greenhouse gas emissions originating from the agriculture sector are examined (see Fig. 1), it is seen that the emissions originating from enteric fermentation, primarily CH_4_, are produced with a rate of 49%, while 39.2% of enteric fermentation originates from cattle, 7% from ovine, and 2.6% from other animals. Enteric fermentation is followed by agricultural soils in the second place with 36% and fertilizer management in the third place with 13% [30].

**Fig. 2:**
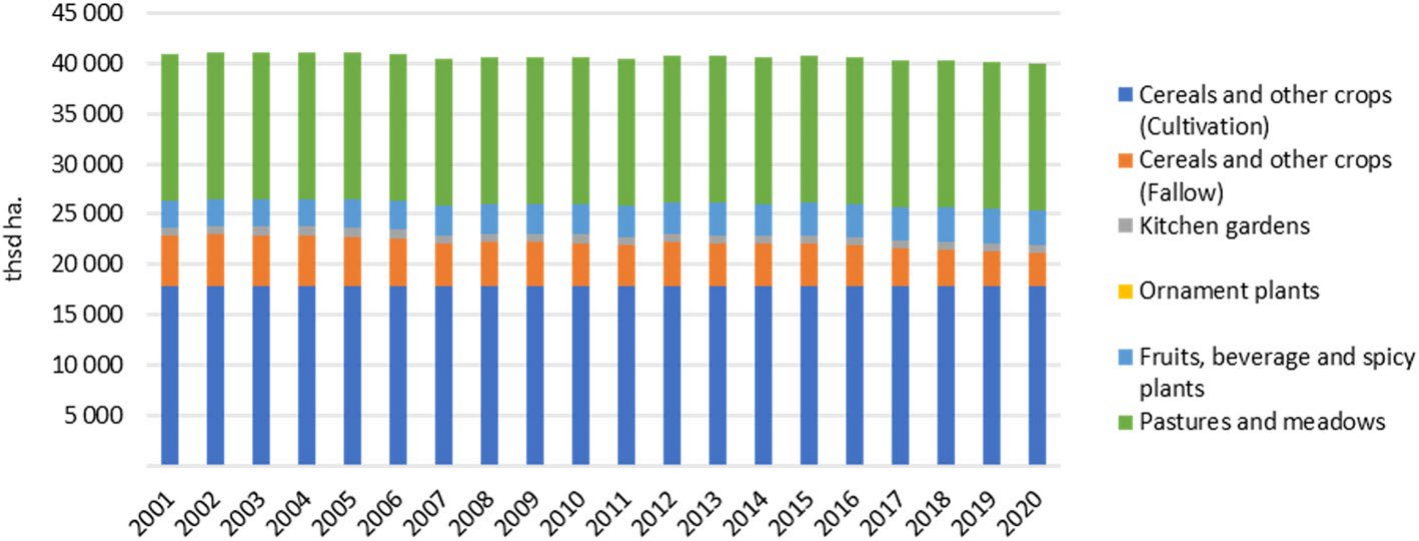
Change in the agricultural lands in Turkey, 2001–2020 (created by the author using TURKSTAT, 2021c data [31])

Greenhouse gas emissions from the agricultural sector have increased significantly especially in the last decade in Turkey. However, the number of agricultural areas has been decreasing recently due to climate change, agricultural malpractices, urbanization, and excessive use of pesticides and chemical fertilizers. While the total agricultural area was 40.9 million hectares in 2001, it decreased to 38.5 million hectares in 2015. The total agricultural area has decreased by 2.4 million hectares, and the cultivated area, which was 17.9 million hectares in 2001, decreased to 15.7 million hectares in 2015 [30, 31]. Considering the change in agricultural area in Turkey, the total agricultural area decreased from 40.9 to 37.8 million hectares between 2001 and 2020; it is observed that meadows and pastures remain constant at 14.6 million hectares (Fig. 2). This is due to the fact that meadow and pasture land data has not been updated after the 2001 General Agricultural Census [31]. In the light of the knowledge of pasture allocations observed within the scope of the study, a question mark arises about the security of data [31]. Hazar (2018) presented that pastures in Turkey are decreasing quantitatively and qualitatively day after day, according to the case studies, personal observations in 50 villages, and in-depth interviews with professionals in her dissertation [19].

Pastures are terrestrial carbon sinks that are important for climate mitigation and the continuity of rural tradition and local sustainable development [32]. Pastures should also be codependently considered with the concept of “food sovereignty,” which has become a crucial agenda to be discussed after the COVID-19 pandemic, as well as the climate crisis. The international farmer movement La Via Campesina describes the basic principles and policies of the concept that ensure food security and food safety. The concept, which was put on the agenda of the United Nations and the UN Food and Agriculture Organization (FAO) in 2008, advocates the rural producers and consumers should cease to be subject to the markets and be democratically involved in production, consumption, and determination policies related to them [33, 34].

However, climate change can reduce productivity and decrease production in crop and animal production, increase food prices, and decrease incomes, which threatens the principles of food sovereignty for various reasons [35]. To prevent this, agroecological farming, as an example of restorative farming methods, has been encouraged in recent years. The concept of “agroecology” has been institutionalized since the 1990s as a science, practice, and field of action. It is an understanding that explains the operation of the agrosystem by using biological, ecological, sociocultural, economic, and political mechanisms, functions, relations, and designs and enabling sustainable agriculture [36]. Any assessment of the adverse effects of climate change on agroecology (e.g., drought, water scarcity, change of crop pattern) should be conducted within the changing socio-economic systems of agricultural conditions. These assessments should focus critically on how the rural population will cope with climate change, including the issue of food sovereignty [37].

Today, sustainable, environmentally friendly, and agroecological food production systems are demanded by modern consumers seeking both quality and safety. It is generally accepted in Turkey that grass-fed animals are healthier than animals fed with corn silage containing GMOs [16, 19]. In this case, it is seen that extensive production is more advantageous than intensive production in terms of animal welfare and food security, although it is expected that there will be less CO_2_ equivalent gas emissions per kg product in sheep farms that adopt the intensive production. With the increasing awareness of producers and consumers in recent years, alternative agri-food initiatives, food networks, and similar common initiatives have emerged. Promoting these initiatives through self-managed and self-sustaining production tools such as local governments, community farming, and farm schools may support agroecological production and food sovereignty [33]. This trend can also contribute to climate change mitigation through an enhanced growth of vegetation in terms of access and allocation of resources and biodiversity protection, while increasing the rural income. The diversity of major farming systems associated with semi-natural habitats and rural landscapes is also crucial for mutually supporting economic sectors such as agriculture, agrotourism, and ecotourism [3, 19, 38].

## Materials and Methods

An approach to reduce the methane (CH_4_) emissions is regulating the enteric fermentation by using more efficient forage crops in husbandry. Emissions can be reduced through special digestive additives and long-term management strategies. Improving grazing conditions and increasing animal productivity are also among the improved feeding practices within the management strategies. Animal health is a crucial factor to improve animal productivity and has many nutritional benefits. Accordingly, grass-fed animals are accepted to be healthier in terms of respiratory, infectious diseases, metabolic stress, and skin and meat quality [29, 39, 40]. Moreover, grazing management has a significant impact on the sustainability of perennial forage crop farming, and organic farming and mass husbandry can cooperatively progress together. As Hazar Kalonya (2018) has established, there are ecosystem tools like a fire tool, a rest tool, a grazing tool, and an animal-effect tool of grazing management that can help to find a symbiosis of mass husbandry and organic farming [19, 29, 41].

In addition, the widespread use of compost in agriculture can improve soil quality and fertility, while also reducing the use of mineral fertilizer. The importance—and thus its potential for improvement of sheep farming in Turkey is given in its numbers: The total number of cattle individuals, including buffalo, culture cattle, cross cattle, and native cattle, is 14.1 million. The total number of sheep and goat individuals, including merino sheep, angora goat, and farm sheep and goats, is 41.9 million (Table 1).

**Table 1:** Number of small cattle, 2015 (adapted from Agacayak and Ozturk, 2017 [29])

Species	Goat	Angora goat	Sheep	Merino sheep	Total
Number	10,210,338	209,228	29,302,358	2,206,300	41,928,224
Waste (ton/year)	6,708,192	274,926	19,251,649	1,449,539	27,684,306

If this waste is composted by optimum composting conditions (carbon/nitrogen: 25–35, humidity 45–60%), it can eliminate the problems caused by animal manure, and the use of mineral fertilizers and related environmental problems can be reduced. It is accepted that the amount of compost to be obtained from the waste of young and adult animals would be in the range of 17–22 million tons. Reduction and adaptation studies can be carried out in order to minimize the effects of climate change on the agricultural sector and to reduce the emissions. The widespread use of compost in agriculture would improve soil quality and fertility and reduce emissions during the production and use of mineral fertilizers [29].

One of the most important criteria of being resistant to climate change, which has serious effects on water and food security, is the reduction of greenhouse gas emissions. Thus, the primary step among the climate change adaptation strategies is to limit and/or reduce the greenhouse gas emissions. Accordingly, emission reduction strategies should be developed and implemented urgently for each sub-sector that causes the emissions [29]. Herein, the vulnerable situation of pastures due to malpractices of the users such as over- and undergrazing, land appropriation to other agricultural or non-agricultural uses, lack of interinstitutional coordination, lack of monitoring, legal gaps, and climate change is a crucial issue.

One effect resulting from climate change can be droughts. A drought experienced in countries such as Turkey, whose main economic sector is agriculture, resulted in the loss of crop and animal products, low product quality, increase in plant and animal diseases, decrease in forage crop yields, limited access to forage crops in husbandry, decrease in the income of rural producers, decrease in agricultural employment, and in parallel, rural–urban migration, lack of raw materials in agriculture-based industries, erosion, and food insecurity. It is seen that various suggestions have been developed to reduce greenhouse gas emissions originating from the agricultural sector and the negative effects of climate change on the agricultural sector in Turkey in the literature [30]. These are the following:

- Protecting water resources by preventing over-irrigation, raising awareness of rural producers, and imposing sanctions.
- Inspecting the use of chemical fertilizers, raising awareness of rural producers, and imposing sanctions in order to reduce greenhouse gas emissions originating from the agricultural sector.
- Providing agricultural credit and insurance facilities to rural producers in drought-affected regions.
- Determining the effects of drought on crop basis and drought-resistant crop varieties.
- Considering climate change while preparing agricultural policies and updating the relevant laws.
- Promoting the use of renewable energy sources in the agricultural sector.
- Ensuring gender equality among rural producers; informing and empowering women producers, especially in agricultural and non-agricultural water management; and encouraging their participation in production.
- Providing trainings on the subject in schools in order to raise environmental/nature awareness in children in urban and rural areas.
- Preventing environmental/nature destruction by preventing the allocation of forest, agricultural, and pasture areas to other uses.
- Ensuring the adaptation of animal production to climate change with better quality feed, improved animal nutrition according to changes in temperature, new stress-resistant animal breeds, effective manure transport and stocking management, grazing management, and pasture improvement studies.
- Diversification and improvement of rangelands, improvement of farm conditions.
- Establishment of cold air chains.
- Extension activities and financing for rural producers.
- It can be summarized as the transition of consumers to local and sustainable food systems, regulation of dietary habits, and improvement of food security.

In addition, regulating enteric fermentation by using more efficient forage crops in husbandry is suggested as an approach to reduce CH_4_ originating from the agricultural sector. Emission reductions can be achieved through improved grazing conditions, improved animal productivity, and special nutritional additives [29]. An option for such additives is anthocyanins or red–orange and lemon extracts [42, 43].

Agacayak and Ozturk (2017) [29] summarize 3 policies and 14 criteria to reduce the greenhouse emissions by directly focusing on the agricultural sector:

1. Enteric fermentation:

- Efficient use of feeds and application of feed additives
- Intensive fattening feed applications
- Addition of certain oils or oil seeds to the food
- Improvement of grazing conditions
- Regulation of protein intake
- Special additives, probiotics

2) Fertilizer management:

- Containment of methane emissions
- Biogas production
- Composting

3) Soil management:

- Soil analysis
- Control of mineral fertilizer applications
- Increasing the soil carbon sequestration capacity with compost applications
- Use of wastes with high carbon content in the soil
- Increasing no-tilling farming practices

Moreover, within the scope of the IPCC [44] emission reduction strategies, the proposed strategies for agriculture and animal husbandry under the 15th title of the United Nations Sustainable Development Goals, “Life on Land,” include promoting the conservation of biological resources through agricultural intensification, reducing deforestation, providing diversity through reclamation and restoration of biodiverse communities on farms and/or pasture lands, and promoting the consumption of vegetable protein rather than animal protein [44].

### Case Study Farms

**Fig. 3:**
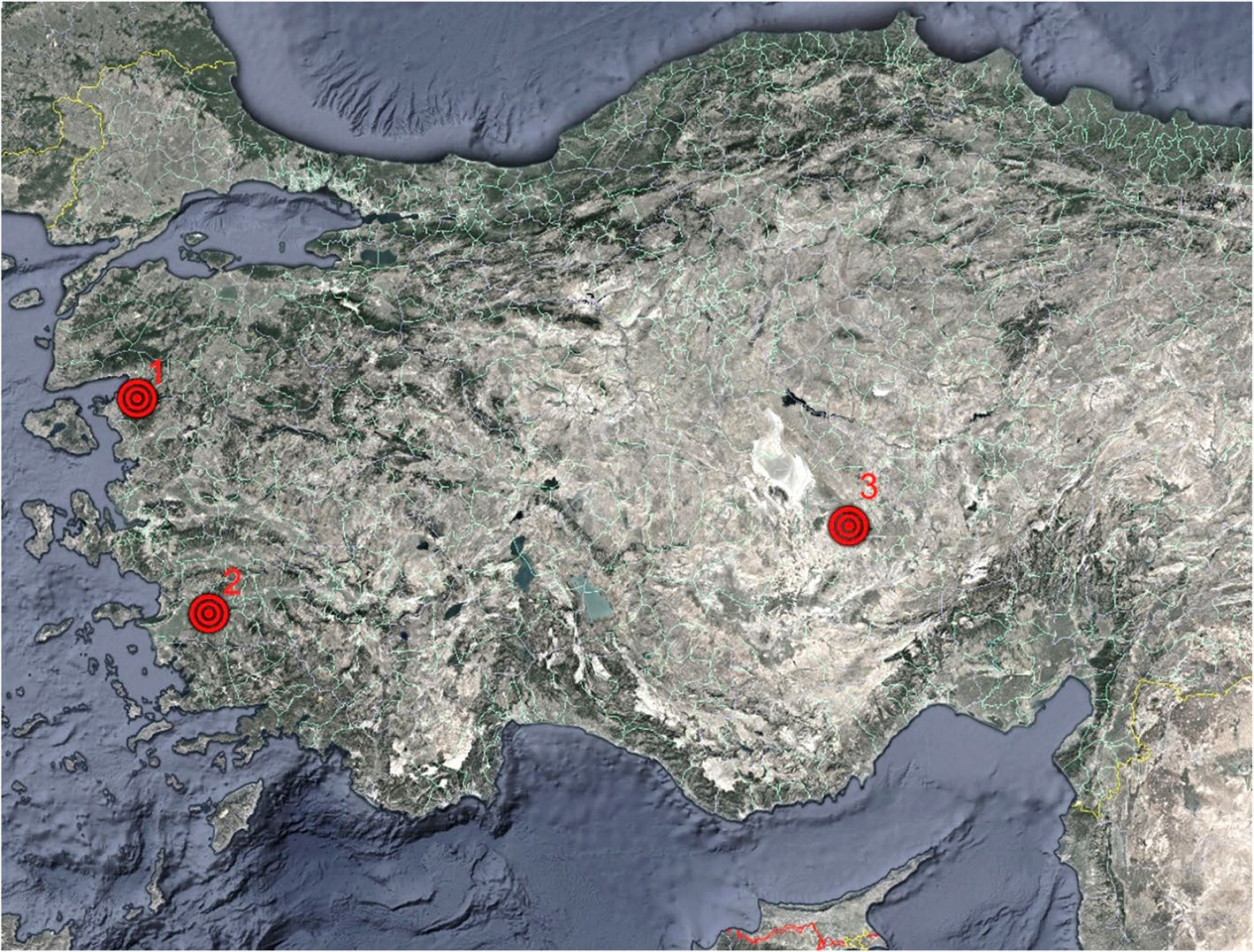
Case study farms in Turkey: (1) Kirtik, Burhaniye, Balikesir; (2) Cincin, Kocarli, Aydin; and (3) Sumru, Taspinar, Aksaray [45]

The animal material of the study was developed for the breeds raised in different climatic regions. In this context, sample breeding systems are chosen in three different breeds and three different bioregions in Turkey (Fig. 3).

The first farm was located at the province of Balikesir in the transitional bioregion of the continental, Black Sea, and Mediterranean. Kivircik breed was used in here as one of the indigenous sheep breeds of Turkey, which has very important meat quality. The second farm was located in Aydin in the Mediterranean bioregion. The Karya breed was used in here as Turkey’s little-known species of the western regions that are crucial in terms of growth properties of lamb. The third farm was located in Aksaray province in the continental bioregion. The Akkaraman breed was used in here, which constitutes the majority of the Turkish sheep population as a fat tail sheep breed (Tables 2 and 3).

**Table 2:** Breeding type and animal material

Breeding type	Akkaraman		Karya		Kivircik		Total
	2019	2020	2019	2020	2019	2020	
Intensive	20	15	20	15	-	15	**85**
Extensive	20	15	17	15	11	15	**93**
Total	**40**	**30**	**37**	**30**	**11**	**30**	**178**

**Table 3:** Animal material and case study locations

Breed	Location	Reason	Climate type
Kivircik	Marmara	Most important breed for the meat quality in Turkish sheep breeds	Transition between the continental, Black Sea, and Mediterranean climates
Karya	Aegean	High reproductive performance, preferred in the western part of Turkey	Mediterranean climate
Akkaraman	Central Anatolia	Majority of the Turkish sheep population	Continental climate

**Fig. 4:**
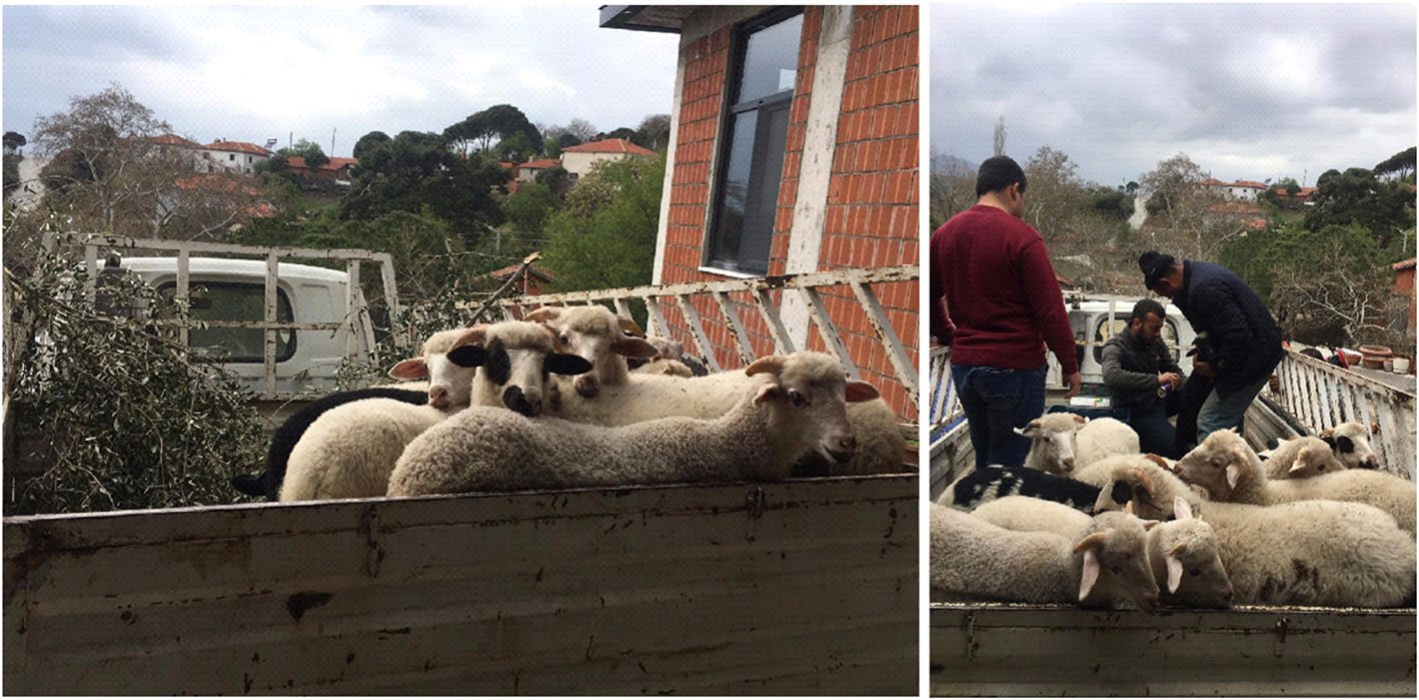
Kirtik Village, Balikesir (transitional bioregion) (personal archive, 2019)

**Fig. 5:**
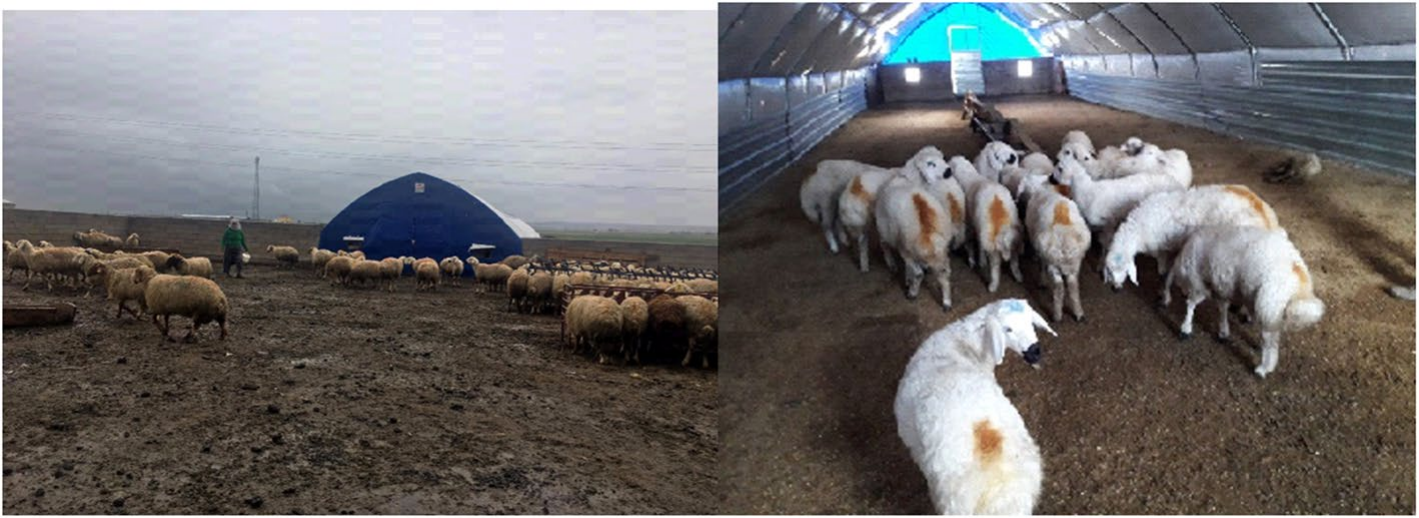
Taspinar Village, Aksaray (continental bioregion) (personal archive, 2019)

In the selection of animal material, male lambs born on similar dates were selected as trial material (Figs. 4 and 5). The lambs constituting the animal material of the experiments were weaned approximately 1 month after birth and divided into two groups as extensive or intensive production. No supplementary feeding was applied to the lambs in the extensive group, and all of the animals were grazed on the pasture. Lamb grower feed obtained from the compound feed factory was given ad libitum to the groups treated with intensive fattening. For example, as forage, alfalfa hay was given as 300 g per lamb per day in grass cages. The fullness and cleanliness of the feeders and drinkers were checked every day. The intensively managed group of lambs was fed barley (42%), corn (24%), soybean meal (10%), wheat bran (4%), molasses (8%), sunflower meal (8%), and vitamin/mineral premix ad libitum with concentrate feed (0.05%), salt (0.95%), and dicalcium phosphate (3%). Concentrated feed given to animals in the intensive group contained 91.0% KM, 13.3% HP, 10.5% HS, 3.2% HY, 55.9% NFE, and 2.550 kcal ME/kg [3].

### Life Cycle Assessment (LCA)

The LCA in this study was conducted in line with the DIN EN ISO 14040 and 14,044 [35, 36]. The approach proposes four steps for a comprehensive evaluation. The first step was the definition of goal and scope. Secondly, a life cycle inventory analysis (LCIA) was to be performed, to define all process stages of the production system and gather the necessary and collectable data. The life cycle inventory (LCI) was finalized by looking for literature data and further information from LCA databases to complete the acquired primary data. Thirdly, the impact assessment for the relevant impact categories was conducted. This study was evaluated for the impact categories global warming potential (GWP), eutrophication potential (EP), and acidification potential (AP), as they reflect the most relevant environmental impacts associated with agricultural systems and combine a global and a local perspective. For this purpose, an LCA model of the production system including all material and energy flows was created. The fourth and final step was the interpretation of the results of the impact assessment [46, 47].

The GaBi Software and databases V8.7 SP40 [48] and herein the CML 2001 methodology including the update in 2016 developed at Leiden University was used to model the production system. The impact assessment method CML2001 restricted quantitative modelling in early steps of the production process to limit uncertainties. The results were grouped into midpoint categories [49].

To ensure comparability, all results were presented in relation to the same amount of product, the functional unit (FU). The assessment of the study was carried out for the FU of 1 kg of lamb meat without bone, in the following termed “kg meat” for readability reasons.

Since it was a challenge to quantify ecosystem services, the LCA of this study also included the impact categories eutrophication potential (EP) and acidification potential (AP). In contrast to the global impact of greenhouse gases in GWP—meaning the worldwide climate change—these two factors indicate the degree of impact of a production process or system on the direct vicinities of the production area—meaning the entry of nutrients on local ecosystems and a changed pH of the soil. Furthermore, a land use analysis (LANCA®) was included, which is described below. This combination of land use, local factors, and GWP presents a holistic analysis of sheep farming and the relevant products, services, and resource emissions along their life cycles.

In order to assess the environmental effects of various lamb meat production systems, three sheep breeding systems (transitional, Mediterranean, and continental bioregions) in Turkey were evaluated using the LCA software GaBi.

**Fig. 6:**
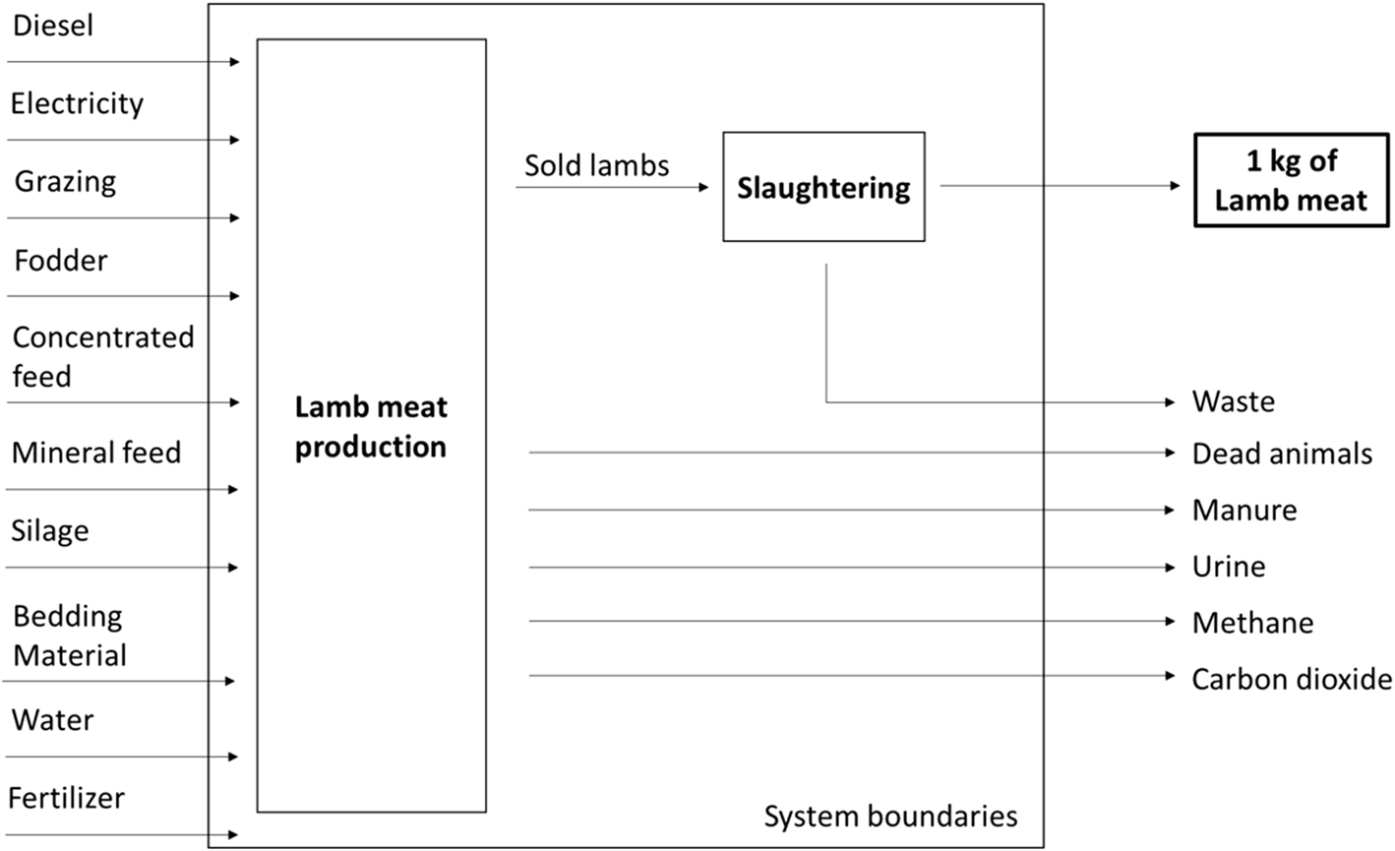
System boundaries with input and output flows (adapted from Geß, et al. 2021 [50])

Figure 6 shows the general system boundaries of a sheep production system.

In order to evaluate the production stages, the amounts stated in the literature (Bollen, 1953; Frohnmayer 2015; Graves 2002) were used for the energy and carbon content of the feed, as well as the emissions by fertilizer, urine, water requirement, ruminating, and respiration, and all effects were evaluated for each manufacturing process [51–53]. These processes were clustered as follows:

- Feed and water (concentrated fodder, mineral, grazing, straw, silage, water, etc.)
- Bedding material
- Respiration and rumination
- Pasture maintenance (manure storage, manure, spreading of manure by machinery, urine emission, etc.)
- Transports (transport to slaughterhouse, manure transport, straw transport, concentrate feed transport, litter transport, etc.)
- Fuel requirement
- Waste treatment (incineration)
- Electricity use
- Thermal energy use
- Auxiliary processes

Since no bedding material was used in the three case study farms and the auxiliary are dummies required for the modelling, these two production processes contained no impacts and are not noted in the results section.

### Land-Use Change Analysis (LANCA®)

The evaluation of the land-use change induced through sheep farming was conducted according to the LANCA® framework recommended by the PEF guidelines [43–45].

**Fig. 7:**
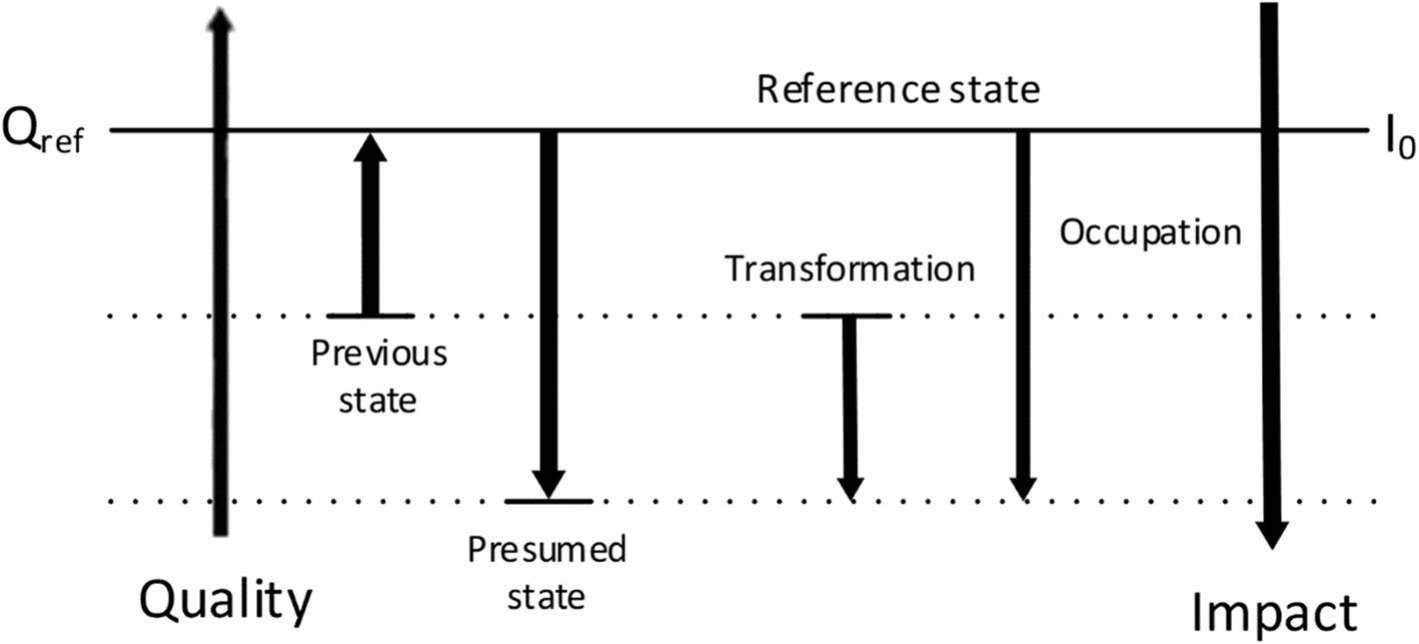
Scheme of the calculation of transformational and occupational effects in the LANCA® methodology (freely adapted from Bos et al., 2016) [55]

LANCA® represented a comparative analysis of the present state of the examined area by quantifying the difference between the present and a fictitious reference state. Furthermore, a distinction was made between transformation and occupation. Transformation effects concern permanent effects caused by the respective land use, which occurred after the land use under consideration. Occupation effects, on the other hand, occurred during the time of the land use under consideration. This approach is depicted in Fig. 7 [54, 55].

The baseline data for the respective reference states of the three case studies directly obtained data was combined with climate datasets from meteorological databases [56]. The input data was completed with the LANCA® background data [54, 55].

#### LCI

In order to carry out the LCA and LANCA, the required LCI data on the land conditions of the farms included in the project was collected, see Table 4 (TUBITAK, 2021) [3]. Furthermore, input data can be found in the supplementary data.

**Table 4:** Inventory data on land conditions used for life cycle assessment (LCA) [3]

		Sümrü/Taşpınar/Aksaray		Kırtık/Burhaniye/Balıkesir		Cincin/Koçarlı/Aydın	
Coordinats							
Longitude:		34°0′42.728″		27°2′21.037″		27°45′14.756″	
Latitude:		38°14′55.975″		39°23′13.682″		37°43′53.634″	
Land use types for all times steps		intensive		extensive		intensive	
		extensive				extensive	
Reference area [m^2^] for one unit		50.000		25.000		56.000	
Duration of land use (occupation) (months)		4		4		4	
Evapotranspiration (mm) (March, April, May & June)		390		391		455	
Effective cation exchange capacity (me. 100 g-1)		** < 2 μm**	**2–50 μm**	** < 2 μm**	**2–50 μm**	** < 2 μm**	**2–50 μm**
		27.9	9.8	46.9	12.8	35.12	14.8
Declination (%)		3		40		2	
Distance groundwater to surface (m);		40		60		20	
Summer precipitation (mm);		35.30		39.80		20.10	
Precipitation (2018–2019) (mm);		346.50		583.20		647.00	
Soil Texture	Clay(%)	31.60		16.8		23.1	
	Silt (%)	41.00		47.1		30.5	
	Sand (%)	27.40		36.1		46.4	
Humus Content	Org. Content (%)	1.70		1.39		2.53	
Skeletal Content		** < 2 μm**	**2–50 μm**	** < 2 μm**	**2–50 μm**	** < 2 μm**	**2–50 μm**
		15.4	58.3	17.9	47.2	18.8	35.9
Crop management information		Virgin		Virgin		Virgin	

## Results

**Fig. 8:**
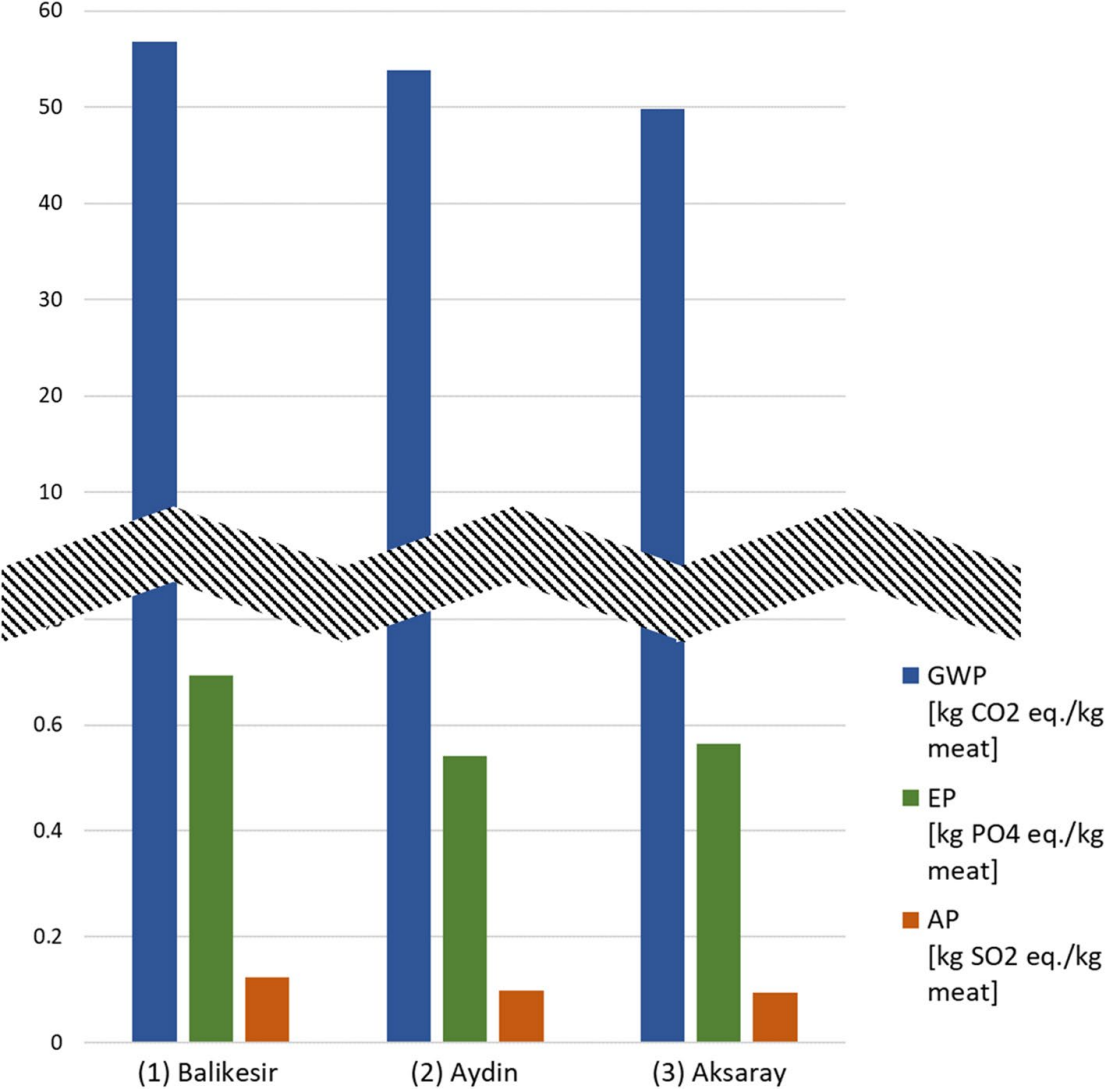
Total LCIA results for the three case study farms (adapted from Geß, et al. 2021 [50])

The LCI data collected from the case study farms were evaluated separately for global warming potential (GWP), eutrophication potential (EP), and acidification potential (AP) impact categories and are presented in Fig. 8 [3].

The three case study farms showed similar GWP results. For the farm in Balikesir, higher EP and AP were calculated. The detailed results are listed in Tables 5, 6, and 7.

**Table 5:** GWP results of all three case study farms (adapted from Geß, et al. 2021 [50])

GWP (kg CO_2_ eq./kg meat)	(1) Balikesir	(2) Aydin	(3) Aksaray
Feed and water	− 72.2	− 43.1	− 45.9
Respiration and rumination	127.8	95.6	94.5
Pasture maintenance	0.04	0.31	0.08
Transport	0.02	0.02	0.04
Fuel demand	0.003	0.048	0.021
Waste incineration	1.4	1.0	1.1
Electricity	− 0.1	0.2	0.0
Thermal energy	− 0.2	− 0.1	− 0.1
Total	**56.8**	**53.9**	**49.8**

**Table 6:** EP results of all three case study farms (adapted from Geß, et al. 2021 [50])

EP (kg PO_4_ eq./kg meat)	(1) Balikesir	(2) Aydin	(3) Aksaray
Feed and water	0.03	0.03	0.04
Respiration and rumination	0.04	0.03	0.03
Pasture maintenance	0.62	0.48	0.49
Transport	0.00002	0.00002	0.00003
Fuel demand	0.000001	0.000015	0.000006
Waste incineration	0.0003	0.0002	0.0002
Electricity	− 0.00004	0.00006	0.00001
Thermal energy	− 0.00002	− 0.00001	− 0.00001
Total	**0.69**	**0.54**	**0.57**

**Table 7:** AP results of all three case study farms (adapted from Geß, et al. 2021 [50])

AP (kg SO_2_ eq./kg meat)	(1) Balikesir	(2) Aydin	(3) Aksaray
Feed and water	0.03	0.02	0.03
Respiration and rumination	0.09	0.07	0.06
Pasture maintenance	0.0003	0.0019	0.0005
Transport	0.0001	0.0001	0.0001
Fuel demand	0.00001	0.0002	0.0001
Waste incineration	0.005	0.003	0.004
Electricity	− 0.0004	0.0006	0.0001
Thermal energy	− 0.0001	− 0.0001	− 0.00004
Total	**0.12**	**0.10**	**0.10**

The process with the largest share of GWP in lamb meat production is the respiration and rumination of the sheep. Since the sheep on these farms are mostly grass-fed, a carbon sequestration can be seen in the production of feed and water. The gains in electric and thermal energy derive from the low consumption and the generation of power and heat through waste incineration.

A different trend than for GWP was observed for EP. Here, pasture maintenance showed the highest impacts. Relevant impact is also given through respiration and rumination and feed and water. Relatively low values are assessed from transport, fuel demand, and waste incineration. The generation of power and heat result in gains or very little emissions of EP for electricity and thermal energy.

The AP analysis results show that in GWP, respiration and rumination have the largest share. Feed and water also show effects on acidification. Transport, fuel demand, and waste incineration show little impacts. Like in EP, the generation of power and heat result in gains or very little emissions of AP for electricity and thermal energy.

In the study, an average of 53.5 kg CO_2_ eq./kg meat for GWP, 0.11 kg SO_2_ eq./kg meat for AP, and 0.60 kg PO_4_ eq./kg meat for EP was calculated.

## Land-Use Change

**Fig. 9:**
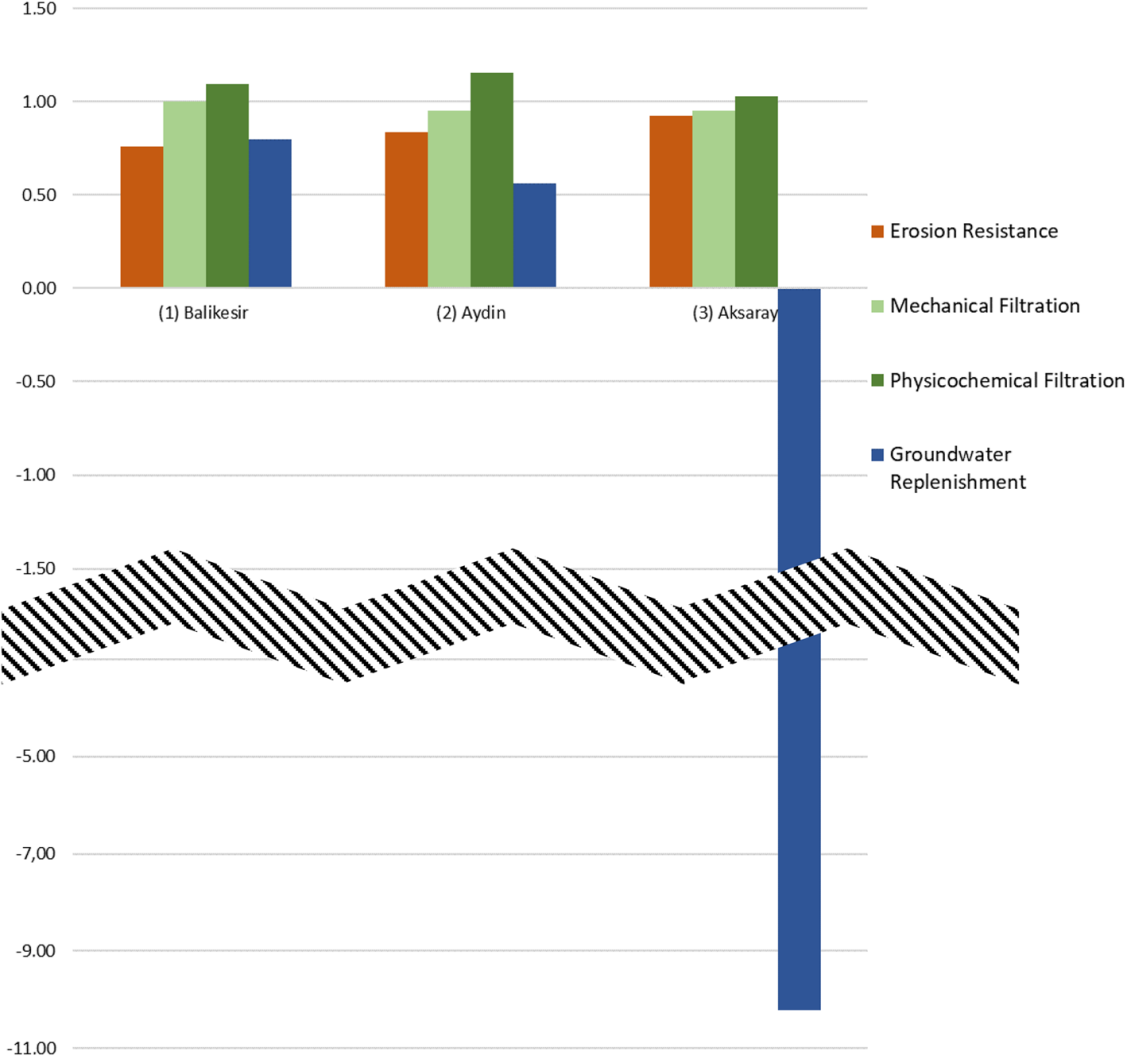
LANCA results for all three case study farms in relation to their respective reference state (adapted from Geß, et al. 2021 [50])

In Fig. 9, the relative land-use change results per impact category for each of the three case study farms are shown. The closer a value is to “1,” the less change is inflicted. A value > 1 indicates an increase, and a factor < 1 indicates a decrease, except for “Erosion Resistance,” which is, by its nature, given in negative values. Therefore, the interpretation is the other way around, and a value > 1 indicates decrease, while a factor < 1 increase.

Figure 9 and Table 8 show that for all farms, an increase in erosion resistance was discovered, the most considerable for the farm in Balikesir. For mechanical filtration and physicochemical filtration, values close to the reference state were assessed. For groundwater replenishment, the highest differences to the reference state were calculated.

**Table 8:** Overall LANCA results for all three case study farms. *PS* present state, *RS* reference state (adapted from Geß, et al. 2021 [50])

	Erosion resistance (t/a)		Mechanical filtration (cm*m^2^/d)		Physicochemical filtration ((cmol*m^2^)/m^2^)		Groundwater replenishment ((mm*m^2^)/a)	
	PS	RS	PS	RS	PS	RS	PS	RS
(1) Balikesir	− 2.01∙ 10^−01^	− 2.64∙ 10^−01^	3.19∙ 10^+07^	3.19∙ 10^+07^	3.85∙ 10^+06^	3.53∙ 10^+06^	3.03∙ 10^+03^	3.79∙ 10^+03^
(2) Aydin	− 4.83∙ 10^−01^	− 5.76∙ 10^−01^	6.80∙ 10^+07^	7.15∙ 10^+07^	1.18∙ 10^+07^	1.02∙ 10^+07^	6.24∙ 10^+03^	1.11∙ 10^+04^
(3) Aksaray	− 1.20∙ 10^−01^	− 1.29∙ 10^−01^	1.21∙ 10^+07^	1.28∙ 10^+07^	1.28∙ 10^+07^	1.24∙ 10^+07^	− 1.26∙ 10^+03^	1.23∙ 10^+02^

## Discussion

The calculated LCA results for the case study farms are to be classified in the upper spectrum of the literature data for LCA results of lamb production systems [50, 57–59].

As the number of lambs born per ewe increases, the relevant GWP is expected to be lower. Under normal conditions, it is expected that there will be less direct CO_2_ equivalent gas emissions per kg product in sheep farms that adopt the intensive production model. However, it is shown that extensive sheep farms have a significantly lower impact in terms of local impact factors like eutrophication and acidification [3, 50]. In this respect, the findings regarding EP for breeds in extensive conditions in the trial conducted in Turkey were lower than those of other sheep production systems in Europe [11, 50]. In Fig. 9 and Table 8, it can be seen that the sheep production system generally showed a low impact on the land-use change. Due to the arid nature of all regions, groundwater replenishment was strongly affected through the water uptake of the sheep. Furthermore, extensively managed grazing sheep even have a positive influence on the surrounding ecosystem and its biodiversity, and as stated above, secondary effects like an enhanced growth of vegetation can lead to subsequent greenhouse gas mitigation [60].

It must be stated that a fault in measurement is suspected: The relation graph in Fig. 9 shows that 10.22 times as much water is taken from the aquifer as is added through natural precipitation. However, if the LANCA® default values for precipitation are entered for the case study area in Aksaray, the results for groundwater replenishment are similar as the ones from the other two case study farms. Hereby, a relation of present to reference state of 0.72 would be calculated. Therefore, it is suspected that the precipitation was not measured correctly, a seasonal drought has occurred, or other external influences have corrupted the measured data.

Husbandry production is traditionally practiced in a rather extensive manner in Turkey, and sheep production is almost entirely perpetuated with the old traditional methods. Mediterranean bioregions create suitable conditions for extensive production on the pastures between April and November. Yet, the breeds are supported by forage crops in winter (e.g., fodder, clover). It is seen that rural producers tend to transform agricultural lands into forage crop production areas (e.g., corn silage) due to the costs, especially for large ruminant breeding regions. However, small ruminant breeding (sheep and goat farming) has a potential to continue on extensive conditions due to have lower costs for producers, although there are considerably minor incentives from the government.

Studies have shown that when it comes to meat quality, extensive production yields similar results to literature results of intensive production showed [61, 62]. However, rural producers primarily consider the quantity rather than quality, considering the cost effectiveness. It is though predicted that the breeders in Turkey will continue practice extensive production when it comes to sheep production. This situation also affects the fluctuations in feed and meat prices.

## Conclusions

It is seen that the vast majority of greenhouse gas emissions originating from the agricultural sector occur as a result of husbandry practices. Although intensive production is frequently pointed out in the literature to reduce emissions, it is seen that the disadvantageous situation in extensive production is caused by wrong husbandry practices and loss of pasture existence. Pasture areas, which host many plant species, have been providing free forage crops to rural producers since the ancient times. The soil and fertilizer management can be achieved with modern and restorative agricultural practices, as well as traditional right husbandry practices. It is seen that vital contributions to local ecosystems can be made when animal husbandry practices are applied with extensive production in pastures and utilizing the grazing abilities of sheep (animal effect). In order to minimize the resulting greenhouse gas emissions, ecosystem tools and services of holistic grazing management can be utilized [50, 60].

Lamb husbandry can provide a vital contribution to local ecosystems when practiced extensively and the grazing capabilities of sheep are put into use. However, due to the sheep’s rumination and the resulting emissions, a high contribution to greenhouse gas total arises. Thus, ecosystem tools and services of the holistic grazing management should be adequately evaluated for minimizing the negative externalities of the extensive production.

The push factors of the rural areas including the lower income from the agriculture and husbandry sector, inadequate technical and social infrastructure services (e.g., education, health), have been triggering the rural–urban migration and urbanization of the rural areas due to decreasing numbers of livestock as well as declining quality and quantity of pastures and agricultural lands. This situation threatens the rural development and food security in Turkey and can be generalized to similar developing countries and geographies.

Promoting agricultural technologies and education of the farmers and breeders in order to prevent malpractices and promoting holistic production systems are crucial factors to achieve rural development and also adaptation to climate change mitigation strategies in a broader sense. Also, information, training, and extension studies for producers and consumers should be expanded for the sustainability of restorative and modern animal husbandry practices.

Pastures have the potential to provide resistance to and reduce the effects of climate change as important carbon sinks. In particular, restorative agriculture practices such as hand pasture reclamation should be carried out under the supervision of relevant institutions and common organizations such as associations and unions, and pastures should be positioned as an important part of urban and rural green infrastructure systems. The protection and improvement of pastures are also important for the continuity of rural tradition and local implicit knowledge. The organization of rural producers should be encouraged through new community-based (common) organizations, training should be given to producers on modern and restorative agricultural practices, and monitoring-supervision systems should be established.

The study aims to promote the sustainable use of natural resources and assets without compromising the quality, competitiveness, or animal welfare and obtain recommendations for the future husbandry systems and rural development in Turkey. For the sustainability of these effective practices, it is important that rural policies are designed together with rural producers, who would be practitioners of modern and restorative agriculture practices, parallel with the participatory planning approach.

To further assess the ecologically beneficial potential of extensive animal husbandry, more species like goat and cattle, but also browsing pigs and poultry, are to be evaluated. Another point of extension is the inclusion of other products like dairy, wool, or fur. By allocating the emission to a variety of products, the emissions per product would significantly decrease. This situation also represents a more likely assumption of extensive farming system, which often works (or have to work) with multiple products to ensure economic profitability.

## Data Availability

The LCI data of this study are available on request from the corresponding author. The GaBi DB background data and models are property of Sphera Solutions GmbH and are not publically available.
